# Lymphocyte to High-Density Lipoprotein Ratio but Not Platelet to Lymphocyte Ratio Effectively Predicts Metabolic Syndrome Among Subjects From Rural China

**DOI:** 10.3389/fcvm.2021.583320

**Published:** 2021-03-12

**Authors:** Shasha Yu, Xiaofan Guo, GuangXiao Li, Hongmei Yang, Liqiang Zheng, Yingxian Sun

**Affiliations:** ^1^Department of Cardiology, First Hospital of China Medical University, Shenyang, China; ^2^Department of Clinical Epidemiology, Institute of Cardiovascular Diseases, First Hospital of China Medical University, Shenyang, China; ^3^Department of Clinical Epidemiology, Shengjing Hospital of China Medical University, Shenyang, China

**Keywords:** lymphocyte to high-density lipoprotein cholesterol ratio, plate to lymphocyte ratio, metabolic syndrome, predictors, rural

## Abstract

**Background:** This study intended to use two novel inflammatory indicators: lymphocyte to high-density lipoprotein cholesterol ratio (LHR) and platelet to lymphocyte ratio (PLR), to predict newly diagnosed metabolic syndrome (MetS) among subjects from rural Northeast China.

**Methods:** Adult participants without MetS at baseline (*n* = 4,980, age = 52.65 ± 10.21 years; 51.9% men) were originated from the Northeast China Rural Cardiovascular Health Study (NCRCHS). LHR (Q1: ≤1.04; Q2: 1.04–1.35; Q3: 1.35–1.79; Q4: ≥1.79) and PLR (Q1: ≤78.50; Q2: 78.50–107.27; Q3: 107.27–140.00; Q4: ≥140.00) were divided in quartile.

**Results:** After 4.66-year follow-up, 1,194 subjects were diagnosed MetS (cumulative incidence 24.0; 25.8% for female and 22.3% for male, *P* = 0.002). Newly diagnosed MetS had higher value of hemoglobin and platelet count compared to those without MetS. As for LHR, from Q1 to Q4, there were increasing value of waist circumference (WC), serum triglycerides (TG), rates of current smoking and drinking whereas decreasing value of HDL-C. However, for PLR, rates of current smoking and drinking significantly decreased from Q1 to Q4. Similarly, the value of WC and TG showed a decreasing trend. In a logistic regression analysis, after adjusted for possible confounders, LHR [OR (95% CI) Q2: 1.13 (0.86, 1.48); OR (95% CI) Q3: 1.23 (0.94, 1.61); OR (95% CI) Q4: 1.57(1.20, 2.06)] but not PLR was effective predictor of newly diagnosed MetS among rural Chinese.

**Conclusion:** MetS had closed relationship with inflammation among subjects from rural China. As a novel marker of inflammation, LHR but not PLR might be an effective predictor of newly diagnosed MetS and should be widely used in the epidemiological study.

## Introduction

The cluster of metabolic disorders, such as increased blood pressure (BP), elevated abdominal waist, dyslipidemia, and insulin resistance were defined as Metabolic syndrome (MetS) (1, 2). Besides, evidence already proved that MetS is associated with 2-fold higher risk of cardiovascular disease (CVD) and 5-fold higher risk of type 2 diabetes (3). Except for the previous mentioned disease, new evidence revealed that many kinds of cancer, hypothyroidism, and cerebral microbleeds were all associated with MetS (4–6). With development of social economy and modification of lifestyle, increasing trend of MetS was exhibited in both developed and developing areas (7). Furthermore, in recent years, MetS showed a more drastic growth among rural subjects (8, 9). A relatively higher rate of MetS was reported in rural middle-aged Koreans when compared to urban Koreans (39.8 vs. 22.5%, *P* < 0.001). Similarly, our previous cross-sectional study claimed a high prevalence (39.0%) of MetS among rural Northeast Chinese (45.6% for female; 31.4% for male) which was even higher than other urban areas (10). More and more emphasis were put on how to reduce MetS and on how to prevent it more effectively. Therefore, it is imminent to estimate new possible predictors for MetS.

Prothrombotic and pro-inflammatory state are the major characters of MetS and related with increased inflammatory cytokine activity (11). In MetS, the over-nutritional status activates several pro-inflammatory signaling pathways, leading to a condition of chronic low-grade inflammation (12). Hemogram parameters, like lymphocyte, platelet count, and biochemical indexes, like HDL-C, LDL-C, were reported to be markers of inflammation. Lymphocyte to high-density lipoprotein cholesterol ratio (LHR) and platelet to lymphocyte ratio (PLR), as a novel inflammatory marker, were proved to be associated with many cardiovascular risk factors (13–17). To the best of our knowledge, there was no studies aiming to assess the possible predictive effect of LHR and PLR on newly diagnosed MetS among rural subjects from Northeast China. Therefore, in the present study, we intend to estimate whether LHR or PLR might be a novel predictor of MetS.

## Methods

### Study Population

The design and inclusion criteria of the community-based prospective cohort study, named the Northeast China Rural Cardiovascular Health Study (NCRCHS), have been described (10). In all, 11,956 participants older than 35 years were enrolled from three countries in LiaoNing Province (Dawa, Zhangwu, and Liaoyang) between 2012 and 2013. The Ethics Committee of China Medical University approved this study (Shenyang, China AF-SDP-07-1, 0-01). During 2015 and 2017, we invited participants at baseline to attend the follow-up study. In total, 1,256 out of 11,956 subjects were excluded due to a lack of contact information. Ultimately, 10,349 participants (86.6%) finished the follow-up visits (median 4.66 follow-up years). All participants signed the written informed consent. In the present study, we excluded those with MetS at bassline. Ultimately, 4,980 participants in the present study.

### Study Variables

Blood pressure was measured automatically followed the standard criteria using an electronic sphygmomanometer (HEM-907; Omron, Tokyo, Japan). Systolic blood pressure (SBP) more than 140 mm Hg and/or diastolic blood pressure (DBP) more than 90 mm Hg, with or without medication, were defined as hypertension (18). After fasting for at least 12 h, participants were gathered together to take blood samples by trained nurses. Fasting plasma glucose (FPG) and lipid profiles, such as low-density lipoprotein cholesterol (LDL-C), high-density lipoprotein cholesterol (HDLC), total cholesterol and triglyceride, were analyzed enzymatically. The Chronic Kidney Disease Epidemiology Collaboration (CKD-EPI) equation was performed to calculate estimated glomerular filtration rate (eGFR) (19). LHR, PLR, and HDLC were divided according to quartered. LHR four groups: Q1: <1.04; Q2: 1.04–1.35; Q3: 1.35–1.79; Q4: ≥1.79; PLR four groups: Q1: <78.50; Q2: 78.50–107.27; Q3: 107.27–140.00; Q4: ≥140.00. HDLC four groups: Q1: <1.26 mmol/L (number of male: 817; female: 453), Q2: 1.26–1.47 mmol/L (number of male: 572; female: 669), Q3: 1.47–1.73 mmol/L (number of male: 563; female: 674), Q4: ≥1.73 mmol/L (number of male: 634; female: 598). MetS was diagnosed follow the unify criteria from the meeting between several major organizations in 2009 (20). The presence of any three of five risk factors constitutes a diagnosis of metabolic syndrome: (1) Elevated waist circumference (population- and country-specific definitions): ≥90 cm for men; ≥80 cm for women (Asians; Japanese; South and Central Americans); (2) Elevated triglycerides (drug treatment for elevated triglycerides is an alternate indicator): ≥150 mg/dL (1.7 mmol/L); (3) Reduced HDL-C (drug treatment for reduced HDL-C is an alternate indicator): <40 mg/dL (1.0 mmol/L) in men; <50 mg/dL (1.3 mmol/L) in women; (4) Elevated blood pressure (antihypertensive drug treatment in a patient with a history of hypertension is an alternate indicator): Systolic ≥130 and/or diastolic ≥85 mm Hg; (5) Elevated fasting glucose (drug treatment of elevated glucose is an alternate indicator): ≥100 mg/dL.

### Statistical Analysis

Mean values ± standard deviations were used to describe continuous variables, and categorical variables were reported as numbers together with percentages. Parametric (one-way analysis of variance followed by *post-hoc* test using Bonferroni correction) and nonparametric (Kruskal-Wallis followed by Wilcoxon) test were used for comparisons categories as appropriate. We used logistic regression analyses to estimate odds ratio (ORs) and 95% confidence intervals (CIs) for the predictive effect of LHR and PLR on MetS after adjusting for possible confounders. SPSS version 17.0 software was used to calculate all the statistical analyses, and statistical significance was defined as *P* ≤ 0.05.

## Results

### Characters of Subjects According to Quartile of LHR

In Table 1, data showed that subjects with relatively higher value of LHR (Q3 and Q4) were younger than lower value (Q1). There was an increasing trend of male and current smoking from Q1 to Q4 (*P* for trend <0.001). Besides, mean value of WC and triglycerides among Q4 LHR was significantly higher than the other three groups. And the HDL-C value was lower in Q3 and Q4 LHR groups than Q1 and Q2. Inversely, SBP and FPG in Q4 was lower compared to Q1. As for results in hemogram, there were increasing trend from Q1 to Q4 in all indexes except for mean platelet volume and platelet distribution width.

**Table 1:** Characters of subjects according to quartile of lymphocyte to high density lipoprotein ratio.

	**Q1** ** (≤1.04)**	**Q2** ** (1.04–1.35)**	**Q3** ** (1.35–1.79)**	**Q4** ** (≥1.79)**	***P*-value**
Age (years)	53.99 ± 10.32	53.37 ± 10.01	52.44 ± 9.43^*^	52.06 ± 10.30^*^^#^	<0.001
Male gender, *n* (%)	380 (45.8)	394 (47.7)	439 (52.4)	492 (59.3)	<0.001
Current smoking, *n* (%)	248 (29.9)	256 (31.0)	329 (39.3)	386 (46.5)	<0.001
Current drinking, *n* (%)	197 (23.8)	187 (22.6)	209 (25.0)	192 (23.1)	0.705
Ethnicity					0.010
Han	800 (96.5)	793 (96.0)	820 (98.0)	788 (94.9)	
Others^a^	29 (3.5)	33 (4.0)	17 (2.0)	42 (5.1)	
Education status					0.772
Primary school or below	433 (52.2)	402 (48.7)	424 (50.7)	407 (49.0)	
Middle school	323 (39.0)	341 (41.3)	334 (39.9)	336 (40.5)	
High school or above	73 (8.8)	83 (10.0)	79 (9.4)	87 (10.5)	
Physical activity					0.371
Light	280 (33.9)	273 (33.3)	272 (32.7)	296 (36.1)	
Moderate	143 (17.3)	142 (17.3)	172 (20.7)	146 (17.8)	
Severe	402 (48.7)	405 (49.4)	387 (46.6)	378 (46.1)	
Waist circumference (cm)	75.58 ± 7.71	76.76 ± 8.03^*^	78.16 ± 7.95^*^^#^	79.05 ± 8.57^*^^#^^†^	<0.001
Systolic blood pressure (mmHg)	134.08 ± 20.21	132.66 ± 19.22	131.51 ± 17.90^*^	131.69 ± 19.25^*^	0.024
Diastolic blood pressure (mmHg)	79.72 ± 10.94	79.18 ± 10.80	79.09 ± 10.21	78.73 ± 10.60	0.303
Fasting plasma glucose, mmol/L	5.66 ± 1.15	5.61 ± 1.10	5.51 ± 0.91^*^	5.53 ± 0.93^*^	0.014
Serum triglycerides, mmol/L	1.06 ± 1.09	1.12 ± 0.73	1.25 ± 0.79^*^^#^	1.27 ± 0.64^*^^#^	<0.001
High-density lipoprotein cholesterol (HDL-C), mmol/L	1.67 ± 0.32	1.47 ± 0.26^*^	1.34 ± 0.24^*^^#^	1.24 ± 0.28^*^^#^^†^	<0.001
Low-density lipoprotein cholesterol (LDL-C), mmol/L	2.77 ± 0.78	2.78 ± 0.77	2.84 ± 0.71	2.82 ± 0.74	0.259
White blood cell count, 10^9^/L	4.92 ± 1.71	5.56 ± 1.89^*^	6.14 ± 1.97^*^^#^	7.39 ± 3.22^*^^#^^†^	<0.001
Neutrophil count, 10^9^/L	3.14 ± 1.65	3.38 ± 1.61	3.54 ± 1.34^*^	4.17 ± 4.32^*^^#^^†^	<0.001
Lymphocyte count, 10^9^/L	1.38 ± 0.30	1.74 ± 0.32^*^	2.07 ± 0.39^*^^#^	3.50 ± 1.98^*^^#^^†^	<0.001
Red blood cell count, 10^12^/L	4.44 ± 0.51	4.53 ± 0.49^*^	4.57 ± 0.48^*^	4.63 ± 0.61^*^^#^^†^	<0.001
Hemoglobin, g/L	131.76 ± 16.67	134.61 ± 22.42^*^	136.65 ± 16.03^*^^#^	141.98 ± 21.30^*^^#^^†^	<0.001
Platelet count, 10^9^/L	200.53 ± 57.70	201.75 ± 59.35	208.57 ± 60.77^*^^#^	208.68 ± 61.22^*^^#^	0.004
Mean platelet volume, fL	10.80 ± 1.92	10.71 ± 3.34	10.52 ± 1.83^*^	10.31 ± 1.56^*^^#^	<0.001
Platelet distribution width, fL	15.61 ± 1.74	15.62 ± 1.43	15.44 ± 1.50^*^^#^	15.08 ± 1.85^*^^#^^†^	<0.001

* *P < 0.05 vs. Q1*,

# *P < 0.05 vs. Q2*,

† *P < 0.05 vs. Q3*;

a *Including some ethnic minorities in China, such as Mongol and Manchu*.

### Characters of Subjects According to Quartile of PLR

The basic characters of subjects according to quartile of PLR were shown in Table 2. Subjects with the highest value of PLR (Q4) were significantly younger than the relatively lower value of PLR. Besides, there was an apparently decreasing trend of male gender percent from Q1 to Q4. In consistent to LHR, the prevalence of current smoking and drinking decreased in PLR groups from Q1 to Q4. The highest mean values of WC and triglycerides whereas the lowest mean value of HDL-C was among subjects at Q4 PLR. Likely, SBP and FPG decreased from Q1 to Q4. The trends of changes in hemogram indexes in PLR groups were like LHR.

**Table 2:** Characters of subjects according to quartile of platelet to lymphocyte ratio.

	**Q1 (≤78.50)**	**Q2 (78.50–107.27)**	**Q3 (107.27–140.00)**	**Q4 (≥140.00)**	***P*-value**
Age (years)	53.35 ± 10.15	53.34 ± 9.91	53.41 ± 10.20	51.73 ± 9.81^*^^#^^†^	0.001
Male gender, *n* (%)	491 (59.1)	474 (57.2)	392 (46.8)	347 (42.2)	<0.001
Current smoking, *n* (%)	369 (44.4)	355 (42.8)	279 (33.3)	216 (26.2)	<0.001
Current drinking, *n* (%)	230 (27.7)	221 (26.7)	180 (21.5)	154 (18.7)	<0.001
Ethnicity					0.940
Han	798 (96.0)	799 (96.4)	808 (96.4)	795 (96.6)	
Others^a^	33 (4.0)	30 (3.6)	30 (3.6)	28 (3.4)	
Education status					0.575
Primary school or below	425 (51.1)	428 (51.6)	421 (50.2)	391 (47.5)	
Middle school	326 (39.2)	316 (38.1)	338 (40.3)	354 (43.0)	
High school or above	80 (9.6)	85 (10.3)	79 (9.4)	78 (9.5)	
Physical activity					0.389
Light	286 (34.9)	254 (30.8)	291(34.9)	290 (35.5)	
Moderate	147 (17.9)	150 (18.2)	154(18.4)	152 (18.6)	
Severe	386 (47.1)	421 (51.0)	390(46.7)	374 (45.8)	
Waist circumference (cm)	77.70 ± 8.30	78.47 ± 8.18	76.87 ± 7.99^*^^#^	76.53 ± 8.10^*^^#^	<0.001
Systolic blood pressure (mmHg)	133.77 ± 19.21	132.14 ± 18.68	132.09 ± 19.21	131.91 ± 19.58^*^	0.166
Diastolic blood pressure (mmHg)	79.42 ± 10.59	79.07 ± 10.36	79.26 ± 10.79	78.98 ± 10.83	0.839
Fasting plasma glucose, mmol/L	5.57 ± 0.89	5.56 ± 0.97	5.58 ± 0.93	5.60 ± 1.28	0.853
Serum triglycerides, mmol/L	1.19 ± 0.68	1.23 ± 1.14	1.15 ± 0.71^#^	1.12 ± 0.73^#^	0.046
High-density lipoprotein cholesterol (HDL-C), mmol/L	1.44 ± 0.32	1.42 ± 0.32	1.42 ± 0.32	1.45 ± 0.32	0.259
Low-density lipoprotein cholesterol (LDL-C), mmol/L	2.84 ± 0.75	2.79 ± 0.70	2.76 ± 0.72^*^	2.83 ± 0.82	0.151
White blood cell count, 10^9^/L	7.03 ± 2.52	6.19 ± 2.82^*^	5.56 ± 1.40^*^^#^	5.24 ± 2.44^*^^#^^†^	<0.001
Neutrophil count, 10^9^/L	4.02 ± 1.47	3.56 ± 1.65^*^	3.37 ± 1.17^*^	3.29 ± 1.43^*^^#^	<0.001
Lymphocyte count, 10^9^/L	3.39 ± 0.50	2.08 ± 0.48^*^	1.75 ± 0.37^*^^#^	1.46 ± 0.35^*^^#^^†^	<0.001
Red blood cell count, 10^12^/L	4.65 ± 0.63	4.60 ± 0.51^*^	4.51 ± 0.46^*^^#^	4.42 ± 0.48^*^^#^^†^	<0.001
Hemoglobin, g/L	144.11 ± 19.48	138.46 ± 15.05^*^	133.84 ± 22.79^*^^#^	128.56 ± 16.95^*^^#^^†^	<0.001
Platelet count, 10^9^/L	162.28 ± 53.54	191.72 ± 43.05^*^	213.65 ± 44.36^*^^#^	252.28 ± 58.09^*^^#^^†^	<0.001
Mean platelet volume, fL	10.10 ± 1.16	10.35 ± 3.32^*^	10.82 ± 1.98^*^^#^	11.06 ± 1.97^*^^#^^†^	<0.001
Platelet distribution width, fL	14.64 ± 1.96	15.65 ± 1.48^*^	15.78 ± 1.23^*^	15.69 ± 1.58^*^	<0.001

* *P < 0.05 vs. Q1*,

# *P < 0.05 vs. Q2*,

† *P < 0.05 vs. Q3*;

a *Including some ethnic minorities in China, such as Mongol and Manchu*.

### Cumulative Incidence of MetS Among Different Groups of PLR and LHR and the Mean Value of PLR and LHR Among Different Numbers of Metabolic Disorders

**Figure 1:**
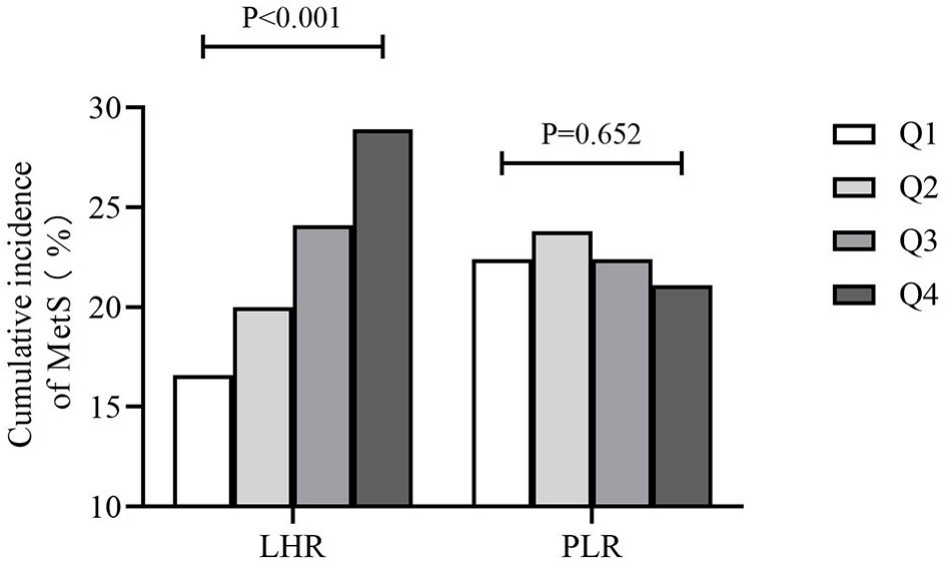
Cumulative incidence of MetS during the 4.66-year follow-up. MetS, metabolic syndrome; PLR, platelet to lymphocyte ratio; LHR, lymphocyte to high density lipoprotein ratio.

**Figure 2:**
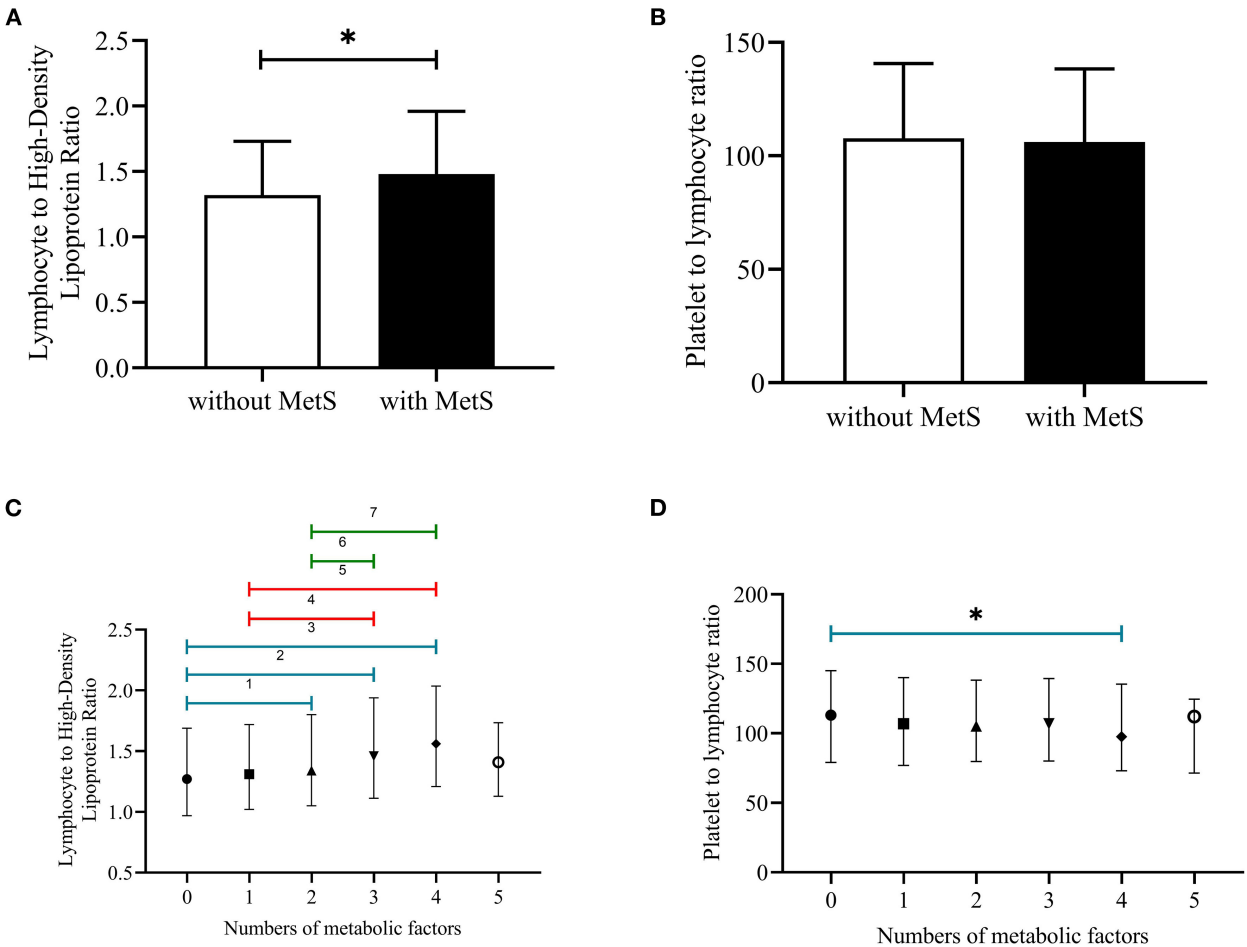
**(A–D)** Median with interquartile range of LHR and PLR among subjects with or without MetS and different numbers of metabolic disorders. PLR, platelet to lymphocyte ratio; LHR, lymphocyte to high density lipoprotein ratio; ^1^*P* < 0.05, no metabolic factors vs. two metabolic factors; ^2^*P* < 0.05, no metabolic factors vs. three metabolic factors; ^3^*P* < 0.05, no metabolic factors vs. four metabolic factors; ^4^*P* < 0.05, one metabolic factor vs. three metabolic factors; ^5^*P* < 0.05, one metabolic factors vs. four metabolic factors; ^6^*P* < 0.05, two metabolic factors vs. three metabolic factors; ^7^*P* < 0.05, two metabolic factors vs. four metabolic factors; *P < 0.05, no metabolic factor vs. four metabolic factors.

Figure 1 showed that the cumulative incidence of MetS during the 4.66 years follow-up was 24.0%. With the increase of LHR value (from Q1 to Q4), the incidence of MetS showed a significant increase (*P* < 0.001). The rates of MetS among different LHR groups were 16.6% in Q1, 20.0% in Q2, 24.1% in Q3, and 28.9% in Q4. As for PLR, there was lack of statistical difference between different groups (*P* = 0.652). Figure 2 showed that there was significant different value of LHR and PLR between subjects with or without MetS (A, B) and between different numbers of metabolic disorder (C, D). Subjects with Mets had significantly higher value of LHR compared to subjects without MetS. Data showed that with increased numbers of metabolic disorders, the value of LHR also increased. However, there was lack of difference between five metabolic disorders group and other groups. As for PLR in different numbers of metabolic disorders, the value of PLR only differed between 0 metabolic disorders and 4 metabolic disorders.

### Baseline Characters of Subjects According to Metabolic Syndrome Status at Follow-Up

Baseline characters of newly diagnosed MetS were showed in Table 3 by gender. Newly diagnosed MetS had significantly higher value of ages, hemoglobin, platelet count, anthrometric parameters, BP, and lipid profiles than subjects without it. Besides, MetS subjects had relatively higher rates of primary school or below education level and light physical activity than their counterparts.

**Table 3:** Baseline characters of subjects according to metabolic syndrome status at follow-up.

	**All in total**			**MetS(–)**			**Newly onset MetS(+)**			**Total-*P*-value**
	**Total** ** (*n* = 4,980)**	**Male** ** (*n* = 2,586)**	**Female** ** (*n* = 2,394)**	**Total** ** (*n* = 3,786)**	**Male** ** (*n* = 2,010)**	**Female** ** (*n* = 1,776)**	**Total** ** (*n* = 1,194)**	**Male** ** (*n* = 576)**	**Female** ** (*n* = 618)**	
Age (years)	52.65 ± 10.21	54.26 ± 10.55	50.91 ± 9.54^*^	52.20 ± 10.28	54.20 ± 10.63	49.93 ± 9.35^*^	54.08 ± 9.89	54.47 ± 10.24	53.72 ± 9.54	<0.001
Current smoking, *n* (%)	1,928 (38.7)	1,536 (59.4)	392 (16.4)^*^	1,486 (39.2)	1,212 (60.3)	274 (15.4)^*^	442 (37.0)	324 (73.3)	118 (19.1)^*^	0.089
Current drinking, *n* (%)	1,278 (25.7)	1,207 (46.7)	71 (3.0)^*^	970 (25.6)	918 (45.7)	52 (2.9) ^*^	308 (25.8)	289 (93.8)	19 (6.2)^*^	0.486
Ethnicity			0.381			0.460			0.405	0.216
Han	4,664 (93.7)	2,425 (93.8)	2,239 (93.5)	3,552 (93.8)	1,887 (93.9)	1,665 (93.8)	1,112 (93.1)	538 (93.4)	574 (92.9)	
Others ^a^	316 (6.3)	161 (6.2)	155 (6.5)	234 (6.2)	123 (6.1)	111 (6.3)	82 (6.9)	38 (6.6)	44 (7.1)	
Education status				<0.001		<0.001			<0.001	0.002
Primary school or below	2,331 (46.8)	1,090 (42.2)	1,241 (42.2)	1,720 (45.4)	845 (42.0)	875 (49.3)	611 (51.2)	245 (42.5)	366 (59.2)	
Middle school	2,167 (43.5)	1,237 (47.8)	930 (38.8)	1,689 (44.6)	964 (48.0)	725 (40.8)	478 (40.0)	273 (47.4)	205 (33.2)	
High school or above	482 (9.7)	259 (10.0)	223 (9.3)	377 (10.0)	201 (10.0)	176 (9.9)	105 (8.8)	58 (10.1)	47 (7.6)	
Physical activity			<0.001			<0.001			<0.001	0.002
Light	1,537 (31.2)	658 (25.7)	879 (37.1)	1,123 (29.9)	508 (25.5)	615 (35.0)	414 (35.1)	150 (26.5)	264 (43.1)	
Moderate	918 (18.6)	458 (17.9)	460 (19.4)	721 (19.2)	368 (18.4)	353 (20.1)	197 (16.7)	90 (15.9)	107 (17.5)	
Severe	2,479 (50.2)	1,446 (56.4)	1,033 (43.5)	1,910 (50.9)	1,119 (56.1)	791 (45.0)	569 (48.2)	327 (57.7)	242 (39.5)	
Waist circumference (cm)	78.39 ± 8.38	80.22 ± 7.93	76.43 ± 8.41^*^	77.28 ± 8.06	79.05 ± 7.45	75.27 ± 8.26^*^	81.94 ± 8.40	84.29 ± 8.23	79.75 ± 7.97^*^	<0.001
Systolic blood pressure (mmHg)	136.55 ± 21.85	139.91 ± 21.83	132.92 ± 21.29^*^	134.58 ± 21.05	138.1 ± 21.26	130.51 ± 20.04^*^	142.79 ± 23.16	145.95 ± 22.72	139.84 ± 23.20^*^	<0.001
Diastolic blood pressure (mmHg)	79.73 ± 11.00	81.52 ± 11.08	77.80 ± 10.59^*^	79.92 ± 10.69	80.65 ± 10.69	76.95 ± 10.35^*^	82.33 ± 11.58	84.55 ± 11.90	80.25 ± 10.89^*^	<0.001
Fasting plasma glucose, mmol/L	5.50 ± 1.06	5.63 ± 1.19	5.37 ± 0.88^*^	5.47 ± 1.03	5.60 ± 1.21	5.33 ± 0.75^*^	5.60 ± 1.14	5.71 ± 1.10	5.50 ± 1.17^*^	<0.001
Serum triglycerides, mmol/L	1.14 ± 0.75	1.17 ± 0.89	1.19 ± 0.56^*^	1.07 ± 0.55	1.09 ± 0.63	1.03 ± 0.45^*^	1.36 ± 1.14	1.45 ± 1.43	1.28 ± 0.78^*^	<0.001
High-density lipoprotein cholesterol (HDL-C), mmol/L	1.52 ± 0.39	1.51 ± 0.42	1.54 ± 0.34^*^	1.55 ± 0.39	1.53 ± 0.42	1.56 ± 0.34	1.46 ± 0.37	1.44 ± 0.42	1.47 ± 0.32	<0.001
Low-density lipoprotein cholesterol (LDL-C), mmol/L	2.83 ± 0.76	2.82 ± 0.74	2.83 ± 0.78	2.75 ± 0.73	2.76 ± 0.72	2.74 ± 0.75	3.06 ± 0.80	3.02 ± 0.77	3.11 ± 0.83	<0.001
White blood cell count, 10^9^/L	6.41 ± 1.56	6.48 ± 1.41	6.32 ± 1.69	6.33 ± 1.52	6.52 ± 1.60	6.12 ± 1.50	6.45 ± 1.57	6.34 ± 1.73	6.93 ± 2.17	0.545
Neutrophil count, 10^9^/L	3.56 ± 2.57	3.75 ± 2.88	3.36 ± 2.17^*^	3.55 ± 2.63	3.74 ± 2.92	3.35 ± 2.25^*^	3.58 ± 2.35	3.77 ± 2.72	3.40 ± 1.91^*^	0.785
Lymphocyte count, 10^9^/L	2.17 ± 2.63	2.25 ± 3.09	2.09 ± 2.04	2.17 ± 2.81	2.24 ± 3.21	2.10 ± 2.30	2.17 ± 1.91	2.29 ± 2.60	2.05 ± 0.79	0.962
Red blood cell count, 10^12^/L	4.60 ± 1.24	4.81 ± 1.25	4.36 ± 1.19^*^	4.58 ± 1.27	4.80 ± 1.39	4.33 ± 1.05^*^	4.65 ± 1.17	4.87 ± 0.49	4.44 ± 1.52^*^	0.112
Hemoglobin, g/L	138.79 ± 19.69	147.99 ± 19.28	128.89 ± 14.69^*^	138.35 ± 19.84	147.20 ± 19.24	128.38 ± 15.24^*^	140.19 ± 19.17	150.79 ± 19.19	130.34 ± 12.86^*^	0.005
Plate count, 10^9^/L	212.41 ± 64.24	203.13 ± 67.52	222.39 ± 58.93^*^	210.81 ± 65.68	202.89 ± 70.55	219.72 ± 58.47^*^	217.49 ± 59.21	203.95 ± 55.71	230.04 ± 59.63^*^	0.002
Mean plate volume, fL	10.88 ± 3.08	10.82 ± 3.09	10.94 ± 3.08	10.88 ± 3.15	10.86 ± 3.38	10.90 ± 2.87	10.85 ± 2.86	10.67 ± 1.68	11.03 ± 3.62^*^	0.782
Plate distribution width, fL	14.78 ± 3.50	14.74 ± 3.42	14.82 ± 3.58	14.76 ± 3.11	14.64 ± 2.05	14.89 ± 3.97^*^	14.84 ± 4.53	15.08 ± 6.15	14.61 ± 2.08	0.476

a *Including some ethnic minorities in China, such as Mongol and Manchu*.

* *P <0.05 compared with counterpart group*.

### Logistic Regression Analysis Showing Independent Predictors of Newly Diagnosed MetS

In Table 4, we can see that after adjusted for age, gender, race, current smoking, current drinking, physical activity intensity, educational status, BMI, LDL-C, TC, eGFR, LHR, and HDLC were both correlated with newly diagnosed MetS. The highest value groups of LHR had significant increased risk of developing MetS [Q4: 1.57 (1.20, 2.06)]. Likewise, compared with higher value of HDLC (Q4), the lowest HDLC level was associated with higher possibility to get MetS [Q1: 1.86 (1.28, 2.72)]. However, after subdivided by gender, we could see that LHR was still able to predict newly diagnosed MetS among both male [Q4:1.52 (1.02, 2.26)] and female [Q4:1.55 (1.06, 2.28)] whereas the predictive effect of HDLC only worked in female [Q1:2.08 (1.21, 3.58)] but not male subjects [Q4:1.71 (0.99, 2.94)]. PLR was not able to predict newly diagnose MetS among rural subjects.

**Table 4:** Binomial logistic regression analysis showing independent predictors of newly diagnosed MetS.

	**Model 1 [OR (95% CI)]**			**Model 2 [OR (95% CI)]**			**Model 3 [OR (95% CI)]**		
	**Total**	**Male**	**Female**	**Total**	**Male**	**Female**	**Total**	**Male**	**Female**
**PLR**
Q1	1.00 (refer)	1.00 (refer)	1.00 (refer)	1.00 (refer)	1.00 (refer)	1.00 (refer)	1.00 (refer)	1.00 (refer)	1.00 (refer)
Q2	1.08 (0.86, 1.36)	1.08 (0.86, 1.36)	1.27 (0.90, 1.79)	1.09 (0.86, 1.37)	0.95 (0.69, 1.29)	1.34 (0.94, 1.90)	1.07 (0.83, 1.37)	0.92 (0.66, 1.29)	1.34 (0.91, 1.96)
Q3	1.00 (0.80, 1.26)	1.00 (0.80, 1.26)	1.04 (0.74, 1.45)	0.99 (0.78, 1.25)	0.95 (0.69, 1.32)	1.09 (0.77, 1.53)	1.05 (0.81, 1.35)	0.98 (0.69, 1.40)	1.19 (0.82, 1.72)
Q4	0.93 (0.74, 1.17)	0.93 (0.74, 1.17)	0.98 (0.70, 1.37)	0.92 (0.73, 1.17)	0.83 (0.59, 1.17)	1.11 (0.79, 1.57)	0.97 (0.75, 1.26)	0.87 (0.60, 1.27)	1.15 (0.79, 1.68)
**LHR**
Q1	1.00 (refer)	1.00 (refer)	1.00 (refer)	1.00 (refer)	1.00 (refer)	1.00 (refer)	1.00 (refer)	1.00 (refer)	1.00 (refer)
Q2	**1.25 (0.97, 1.61)**	**1.10 (0.75, 1.61)**	**1.39 (1.00, 1.93)**	**1.31 (1.01, 1.68)**	1.15 (0.78, 1.70)	**1.44 (1.03, 2.02)**	1.13 (0.86, 1.48)	0.97 (0.64,1.48)	1.26 (0.88, 1.81)
Q3	**1.59 (1.25, 2.03)**	**1.70 (1.19, 2.43)**	**1.53 (1.10, 2.13)**	**1.71 (1.33, 2.18)**	**1.82 (1.27, 2.62)**	**1.55 (1.11, 2.18)**	1.23 (0.94, 1.61)	1.26 (0.84,1.89)	1.12 (0.77,1.63)
Q4	**2.04 (1.61, 2.58)**	**2.12 (1.51, 2.99)**	**2.05 (1.47, 2.86)**	**2.28 (1.79, 2.91)**	**2.37 (1.67, 3.37)**	**2.22 (1.57, 3.13)**	**1.57 (1.20, 2.06)**	**1.52 (1.02, 2.26)**	**1.55 (1.06, 2.28)**
**HDLC**
Q4	1.00 (refer)	1.00 (refer)	1.00 (refer)	1.00 (refer)	1.00 (refer)	1.00 (refer)	1.00 (refer)	1.00 (refer)	1.00 (refer)
Q1	**1.96 (1.51, 2.56)**	**2.26 (1.54, 3.34)**	**1.75 (1.20, 2.55)**	**2.44 (1.85, 3.23)**	**2.83 (1.88, 4.27)**	**2.28 (1.54, 3.38)**	**1.86 (1.28, 2.72)**	1.71 (0.99, 2.94)	**2.08 (1.21, 3.58)**
Q2	**1.60 (1.21, 2.10)**	**1.45 (0.95, 2.22)**	**1.69 (1.18, 2.43)**	**1.75 (1.32, 2.32)**	**1.64 (1.07, 2.54)**	**1.95 (1.34, 2.83)**	1.32 (0.94, 1.84)	1.16 (0.70, 1.93)	1.45 (0.92, 2.29)
Q3	1.07 (0.80, 1.03)	1.08 (0.69, 1.70)	1.05 (0.71, 1.55)	1.13 (0.84, 1.52)	1.19 (0.76, 1.89)	1.12 (0.75, 1.67)	0.91 (0.65, 1.03)	0.99 (0.60, 1.04)	0.86 (0.55, 1.07)

*Model 1: Not adjusted; Model 2: Adjusted for age, gender, race, current smoking, current drinking, physical activity intensity, educational status. Model 3: Adjusted for age, gender, race, current smoking, current drinking, physical activity intensity, educational status, BMI, LDL-C, TC, eGFR, WC, FPG, SBP, DBP, TG; PLR, platelet to lymphocyte ratio; LHR, lymphocyte to high density lipoprotein ratio; HDLC, high-density lipoprotein cholesterol; Data in bold means P < 0.05*.

## Discussion

The present study revealed that subjects with MetS had higher LHR values when compared to subjects without MetS. Besides, there was a graded relationship between increasing numbers of metabolic disorders and LHR. Increasing value of LHR but not PLR, was significantly and positively correlated with higher incidence of MetS among subjects from rural China. To the best of our knowledge, our study for the first time, focused on the relationship between LHR as a novel predictor of inflammation and the newly diagnosed MetS among rural subjects.

The rate of MetS varied due to the different definitions. Even so, the relatively higher rate of MetS still showed the grim situation. MetS became more and more prevalent among both developed and developing areas. A national study in Iran in 2007 exhibited a high rate of MetS with 34.7% based on ATP III criteria, 37.4% based on IDF definition, and 41.6% based on ATP III/AHA/NHLBI criteria (21). Similarly, there were other studies reported that the incidence of MetS varied from 16.2 to 37.1% around the world (22–25). MetS was proved to be associate with cardiovascular disease and cardiovascular mortality. Previous Meta-analysis which included 87 studies (951,083 subjects), concluded that MetS had a 2-fold increase in cardiovascular outcomes and a 1.5-fold increase in all-cause mortality (26). There were many mechanisms related with cardiovascular disease in MetS. First is central obesity and insulin resistance. Obesity could induce chronic low-grade inflammation and cause hypoxia, oxidative stress and endoplasmic reticulum (27). Pro-inflammatory factors, like IL-6, TNF-α, MCP-1, also aggravated glucose dysregulation and inhibited normal insulin signaling, resulting in insulin resistance (IR) (28). There were multiple studies demonstrated that IR could result in cardiovascular disease *via* pathophysiological mechanisms including, elevated vascular tone and pro-thrombotic state (29, 30). Second is impaired glucose metabolism. Hyperglycemia can evoke oxidative stress, accelerate advanced glycation end products formation and inflammatory response in the vascular system (31, 32). Third is the prothrombotic state. Previous studies confirmed that pro-thrombotic and pro-inflammatory factors could cause the imbalance of coagulation-fibrinolysis which resulted in growing risk of CVD (33, 34). From the mentioned mechanisms above, it is obvious that inflammation serves a central role in the pathogenesis of MetS. Therefore, inflammatory parameters can be used as a useful predictor of MetS.

As novel indicators of inflammation, LHR and PLR were proved to be associated with MetS. Haishan Chen and colleagues reported that MetS was positively correlated with hemogram indexes, like white blood cell count, lymphocyte count, neutrophil count, red blood cell count, and hemoglobin (16). They also concluded that LHR may be a useful marker of inflammation to assess the presence and severity of MetS (16). Similarly, Tong Chen and colleagues revealed that both LHR (OR: 3.671) and neutrophil to high-density lipoprotein cholesterol ratio (NHR) (OR: 1.728) can predict MetS in females only independent of associated risk factors (17). In consistent with previous studies, data from our study also found that increasing value of LHR was correlated with higher risk of MetS. These associations between blood parameters and MetS might be relevant to insulin resistance. As been proved previously, insulin and insulin growth factors, I and II significantly promote RBC and WBC proliferating (35–37). In addition, chronic inflammation in MetS could induce synthesis of many kinds of cytokines and proteolytic enzymes, and cause disruption of endothelial integrity and functional impairment, leading to an increase in WBCs and their subtypes, like lymphocytes (38, 39). Therefore, the increasing numbers of lymphocyte could act as an index of inflammatory status in MetS. As a result, the elevation of WBC counts might cause chronic and low levels inflammation which resulted in impairment of endothelial function and production of nitric oxide and prostacyclin with resultance of vasoconstriction and hypertension. In addition, inflammatory factors change properties of leukocyte, like increase tendency to adhere to the vascular endothelium which resulted in increased vascular resistance (40, 41). In addition to the role of lymphocytes, HDL-C plays an important role in anti-inflammatory, oxidant, and antithrombotic progress through preventing the migration of macrophages in atherosclerosis and promoting the export of oxidized LDL-C (42, 43).

Previous studies reported that increased platelet counts correlated with insulin resistance and MetS (44, 45). In addition, lower lymphocyte count was associated with increased cardiovascular events (46). Hence, PLR was considered to be as a novel potential inflammatory index which showed a close relationship with adverse outcomes in various cardiovascular diseases (13, 14). However, unlike other previous studies, data in our present study could not confirm the possible predictive effect of PLR on nascent MetS among rural residents. One single center large-scale study claimed that the association between PLR and MetS was based on the correlation between PLR and CRP (42). Therefore, the failure of PLR in predicting Mets might be relevant to the lack of measuring CRP in our study. Second, data in our study showed that there was no significant difference of lymphocytes count between nascent MetS and without MetS. Therefore, the validity of the predictive effect is largely depending on HDL-C level and platelet counts. It is possible that in our study HDL-C has a more close relationship with nascent MetS than platelet counts. Ganesh Jialal et al. announced that, unlike Akboga et al., they cannot confirm the findings that PLR was associated with nascent MetS in their carefully selected patients (42, 47). The attributed this discrepancy to the difference of the enrolled participants. Ganesh Jialal et al. excluded diabetes, ASCVD, smoking, patients with inflammatory diseases and macro-inflammation and concluded that both the platelet count and PLR were not increased in nascent MetS (47). Therefore, the difference of enrolled criterion might also result in a complete different conclusion. Our presents study enrolled rural Northeast residents, the population difference might be responsible for the discrepancy of predictive effect of LHR and PLR on nascent MetS. Further studies were in need to better estimation of the possible predictive effect of PLR on MetS.

## Limitations

The present study has some limitations. First, the findings in the present study could not be generalized to all Chinese around the country since the enrolled subjects came from one province in Northeast China. Second, we lost contact with some enrolled subjects during the follow-up. This could result in bias in the correlation between LHR, PLR, and MetS. Third, there was some participant loss of contact during the follow-up. Is possible that the missing data may cause bias in our results. Third, the association between LHR, PLR, and the incidence of MetS was based on a single blood test, which might result in bias. Fourth, the present study intended to figure out one available, simple, and inexpensive parameters that were effectively to predict nascent MetS among rural Chinese subjects. LHR and PLR are easily accessible markers that are less expensive than other inflammation marker [e.g., cytokines, adipokines, CRP, monocyte chemoattractant protein (MCP)-1]. Therefore, we did not measure CRP and other inflammatory markers.

## Conclusion

In conclusion, this study is the first epidemiological study which confirmed that LHR but not PLR was significantly higher in subjects with MetS and LHR was independently associated with newly diagnosed MetS among subjects from rural Northeast China. LHR can act as a simple and effective predictors of MetS.

## Data Availability Statement

The raw data supporting the conclusions of this article will be made available by the authors, without undue reservation.

## Ethics Statement

The studies involving human participants were reviewed and approved by the Ethics Committee of China Medical University (Shenyang, China AF-SDP-07-1, 0-01). The patients/participants provided their written informed consent to participate in this study.

## Author Contributions

SY contributed to the data collection, analysis, and interpretation. XG and HY contributed to data collection. GL and SY contributed to data analysis. YS contributed to the study conceptions and design. All authors read and approved the final version of the manuscript.

## Conflict of Interest

The authors declare that the research was conducted in the absence of any commercial or financial relationships that could be construed as a potential conflict of interest.
